# Differences and Commonalities in Children with Childhood Apraxia of Speech and Comorbid Neurodevelopmental Disorders: A Multidimensional Perspective

**DOI:** 10.3390/jpm12020313

**Published:** 2022-02-19

**Authors:** Anna Maria Chilosi, Irina Podda, Ivana Ricca, Alessandro Comparini, Beatrice Franchi, Simona Fiori, Rosa Pasquariello, Claudia Casalini, Paola Cipriani, Filippo Maria Santorelli

**Affiliations:** 1 Department of Developmental Neuroscience, IRCCS “Stella Maris Foundation” Scientific Institute, 56128 Pisa, Italy; ivana.ricca@fsm.unipi.it (I.R.); beatrice.franchi@fsm.unipi.it (B.F.); simona.fiori@fsm.unipi.it (S.F.); rosa.pasquariello@fsm.unipi.it (R.P.); claudia.casalini@fsm.unipi.it (C.C.); pcipriani@fsm.unipi.it (P.C.); fsantorelli@fsm.unipi.it (F.M.S.); 2 Parole al Centro Studio di Logopedia, 16129 Genova, Italy; 3 Azienda ASL Toscana Nord Ovest, 56121 Pisa, Italy; alessandro.comparini@uslnordovest.toscana.it

**Keywords:** childhood apraxia of speech, speech and language disorders, comorbidities, complex neurodevelopmental disorders, genetic investigation, chromosome microarray analysis (CMA), neuroimaging

## Abstract

Childhood apraxia of speech (CAS) is a motor speech disorder often co-occurring with language impairment and complex neurodevelopmental disorders. A cohort of 106 children with CAS associated to other neurodevelopmental disorders underwent a multidimensional investigation of speech and language profiles, chromosome microarray analysis and structural brain magnetic resonance (MR). Our aim was to compare the clinical profiles of children with CAS co-occurring with only language impairment with those who, in addition to language impairment, had other neurodevelopmental disorders. Expressive grammar was impaired in the majority of the sample in the context of similar alterations of speech, typical of the core symptoms of CAS. Moreover, children with complex comorbidities also showed more severe and persistent receptive language deficits. About 25% of the participants harbored copy number variations (CNVs) already described in association to neurodevelopmental disorders. CNVs occurred more frequently in children with complex comorbidities. MR structural/signal alterations were found in a small number of children and were of uncertain pathogenic significance. These results confirm that CAS needs multidimensional diagnostic and clinical management. The high frequency of language impairment has important implications for early care and demands a personalized treatment approach in which speech and language goals are consistently integrated.

## 1. Introduction

Childhood apraxia of speech (CAS) is a motor speech disorder, whose core deficit involves the planning and/or programming of the spatiotemporal parameters of speech movement sequences. CAS is defined by the American Speech-Language and Hearing Association [1] as “a neurological childhood disorder, in which the precision and consistency of movements underlying speech are impaired in the absence of neuromuscular deficits (i.e., abnormal reflexes, abnormal tone)”. According to the ASHA consensus, three speech features are characteristic of CAS: (a) inconsistent errors on consonants and vowels during repeated productions of syllables or words; (b) lengthened and disrupted coarticulatory transitions between sounds and syllables; and (c) inappropriate prosody in the realization of lexical or phrasal stress. These features are associated with other speech symptoms [2,3] and may co-occur with persistent language and learning disorders [4,5,6], resulting in an effortful, poorly intelligible speech that negatively impacts the children’s social communication in daily activities, peer interactions and literacy.

Regarding etiology, increasing evidence suggests that CAS has a genetic basis. Mutations in FOXP2, originally described in the multigenerational KE family [7], account for a small proportion of cases [8,9]. The use of chromosome microarray analysis (CMA) and the application of next generation sequencing techniques have denoted a large genetic heterogeneity in the past few years [10,11,12,13,14,15,16] with the identification of gene variants that may implicate shared pathways in broad transcriptional regulation during normal speech development [16,17,18,19,20]. Regarding the neural correlates, routine clinical magnetic resonance (MR) usually does not detect unequivocal causative brain abnormalities in idiopathic CAS [21,22,23].

The clinical subtyping proposed by ASHA [1], according to which children with CAS can be grouped depending on the absence or presence of comorbidities, hints at a complexity and heterogeneity that deserve a more in-depth understanding. In fact, along with its isolated presentation, CAS may co-occur with complex neurodevelopmental disorders such as intellectual disability (ID), attention deficit and hyperactivity disorder (ADHD) and autism spectrum disorder (ASD).

Isolated CAS seems to account for only a part of the cases, while children with complex comorbid profiles represent a large population with different needs in terms of diagnosis, healthcare, treatment and educational support [23]. Furthermore, the co-occurrence of CAS and language impairment (LI) is frequently documented in literature [4,6,22,23,24,25]. In English-speaking children with CAS, some authors described [24,26] the presence of morphological errors that were not entirely explained by the motor speech deficits, thus suggesting a possible linguistic origin. In addition, Lewis et al. [4] reported the persistence of expressive language impairment even after the resolution of the speech symptoms in adolescents with CAS.

The nature of the co-occurrence of CAS and LI is still under debate as it is not clear whether language impairment is coincidental with CAS or if it arises from some common disruptive mechanisms. In a recent paper by Bombonato et al. [27], it was hypothesized that the development of both speech and language may be affected by altered implicit learning skills.

As outlined by Morgan and Webster [23], the evaluation of a child with CAS requires the application of “broader phenotyping approaches” to identify co-occurring disorders and possible etiological correlates. Over the last two decades a perspective shift in the conceptualization of comorbidities has gradually matured, based on the evidence that neurodevelopmental disorders tend to co-occur and to be long-lasting conditions, eventually determining a burden of disability that needs to be quantified early on in development [28].

In the present study, we investigated in a clinical multidimensional perspective a large cohort of Italian children who met the current diagnostic criteria for CAS and for comorbid neurodevelopmental disorders. We considered two groups: the first with only language impairment (CAS-LI) and the second with LI and complex neurodevelopmental comorbidities (CAS-LI + CND). It was assumed that the complex syntactic and morphological structure of Italian, might account for a higher vulnerability of the developing language system in the presence of a severe and persistent primary motor speech disorder. Previous research carried out by our group on a small sample of Italian children with CAS showed the presence of language impairment in most of the children [22]. We were therefore particularly interested in broadening our clinical approach to CAS conceived as a “symptom complex” [29,30] that must be studied considering a wide number of interacting variables. All the children underwent a comprehensive speech/language assessment and first-tier neuroradiological and genetic investigations (structural MR and CMA). This study then compared the clinical profiles of children with CAS co-occurring with only language impairment with the profiles of those with other comorbidities aiming to explore the potential role of different variables on the expressivity of CAS.

## 2. Methods

### 2.1. Participants

A total of 106 consecutive cases were selected from a larger population of about 2000 children attending the neurolinguistic and neuropsychological unit of IRCCS Stella Maris, a tertiary care hospital for children with neurological, neurodevelopmental and psychiatric disorders, over a period of 10 years (2010–2019). Identification of patients with CAS was based on a comprehensive clinical and instrumental assessment (see Section 2.2), which represents the standard clinical protocol adopted by our clinic for the assessment of complex neuropsychological and neurodevelopmental disorders.

Eligibility criteria required Italian as the only or primary language spoken at home, age at clinical evaluation ≥ 4 years and the ability to complete a full neurological and speech and language assessment. Exclusion criteria were orofacial structural abnormalities, audiological deficits, epilepsy, known neurological and neurometabolic disorders and dysarthria.

The diagnosis of CAS was carried out by a multidisciplinary team in accordance with the three ASHA criteria [1] and with any combination of at least five out of the ten Strand’s speech features (see Supplementary Table S1), detectable across three speech contexts that varied in difficulty. The identification of the diagnostic features was based on formal testing and on perceptual analysis of video-recorded speech samples by two independent observers (AC, BF). Language impairment (LI) was diagnosed when a child scored at least −1.5 SD below the mean for age in one or more standardized language tests.

LI occurred in the whole selected sample, while about half of the participants also met the diagnostic criteria for another complex neurodevelopmental disorder (CND). Based on the co-occurrence of only LI or also of other complex comorbidities, 52 children were assigned to the CAS-LI group and 54 children to the CAS-LI + CND group.

Written parental informed consent and child assent for participation in this study and for data publication were obtained in all cases. The study was approved by the ethics committee of the IRCCS Fondazione Stella Maris (Number 13/2013) and by the regional pediatric ethics committee (CEP) 19-03-2018/RF2016-02361560.

### 2.2. Procedures and Measures

#### 2.2.1. Clinical Assessment

All cases underwent standard neurological and psychiatric examination by a child neuropsychiatrist (AC and PC) to diagnose co-occurring neurodevelopmental disorders. Nonverbal IQ was assessed with the Wechsler Preschool and Primary Scale of Intelligence, 3rd Edition (WPPSI-III) or the Wechsler Intelligence Scale for Children, 3rd Edition (WISC-III) and 4th Edition (WISC-IV) depending on the child’s age. Whenever a neurological examination revealed suspected signs of developmental coordination disorder (DCD), the children were also assessed with the Movement ABC-2 Test [31]. A value at or below the fifth percentile was considered as the clinical cut-off score. The diagnosis of ASD and ADHD was carried out by a specialized team according to the DSM-5 [32] clinical diagnostic criteria and using specific assessment procedures for these disorders.

#### 2.2.2. Speech and Language Assessment

To identify the specific characteristics of CAS, including the early signs of the disorder, the assessment protocol included: (a) Parental report on the child’s early vocal behavior, speech, language and early gross motor developmental milestones, as well as familial antecedents for oral/written language disorders; Family history was considered significant if one or more relatives had a history of any type of speech-language or learning disorders, or both; (b) Speech tasks (see Supplementary Table S2 for details) including assessment of phonetic inventory, speech inaccuracy and inconsistency, syllable omissions and diadochokinetic rate (DDK); (c) Perceptual analysis of prosody and intelligibility in spontaneous speech by two independent raters; (d) Analysis of speech characteristics during the administration of speech tasks and in spontaneous production according to Strand’s 10-point checklist (see Supplementary Table S1); (e) Analysis of the level of grammar complexity on speech samples collected during spontaneous verbal interaction and/or the description of a picture story. (f) Administration of standardized tests of receptive and expressive grammar and vocabulary (see Supplementary Table S2). All assessment sessions were videotaped, transcribed and coded independently by two observers (AC, BF).

To estimate the overall level of speech and language proficiency, two composite severity scores were calculated (one for speech and one for language) based on six speech and four language measures. Each measure was assigned a score of zero when normal or borderline and one when deficient. The maximum severity score was six for speech and four for language. The speech composite severity score included phonetic inventory, word inaccuracy and inconsistency of errors, DDK rate, syllable omissions and intelligibility. The language severity score included expressive and receptive vocabulary and grammar.

#### 2.2.3. Genetic Investigations

CMA analyses were performed using the Agilent 8 × 60 K Microarray oligonucleotide platform with a median resolution of 100 Kbp, according to the manufacture’s protocol (Agilent Technologies, Santa Clara, CA, USA). CNV coordinates refer to the Genome Reference Consortium Human Build 37 (GRCh37/hg19). In each proband, CNVs were confirmed by a quantitative polymerase chain reaction (qPCR). Segregation analyses in parental DNA (whenever available) were performed by qPCR. Polymorphic CNVs, based on the Database of Genomic Variants data (DGV) [33]), were filtered out.

Nonpolymorphic CNVs were classified as “causative” (C-CNVs) or “noncausative” (N-CNVs) according to the American College of Medical Genetics and Genomics (ACMG) guidelines [34]. We considered as “causative” in reference to CAS: (1) CNVs encompassing genomic regions or genes associated with CAS, speech and language disorders or with other neurodevelopmental conditions (i.e., intellectual disability, epilepsy, autism) in the Online Mendelian Inheritance in Man (OMIM) database [35] or in the Simons Foundation Autism Research Initiative (SFARI) Gene database [36]; (2) CNVs involving genes reported in association with neurodevelopmental disorders in literature; (3) Abnormalities involving large chromosomal regions (>1.5 Mbp). Conversely, CNVs were considered noncausative (N-CNVs) in reference to CAS if: (1) They have never been associated with CAS, speech and language disorders or with other neurodevelopmental disorders; (2) They involve genes that are not associated with any neurological pathology or genes that are not expressed in the central nervous system (CNS); (3) They encompass chromosomal bands that do not contain any gene. Children who tested negative for CNVs were classified as “without CNVs” (w-CNVs).

#### 2.2.4. Neuroradiological Investigation

Structural brain MR was performed using a 1.5 Tesla MR scanner (GE, Signa Horizon 1.5, Milwaukee, WI, USA). As standard MR protocol, we analyzed T1, T2, T2* planar (2D) or tridimensional (3D) images with spin echo (SE), fast spin echo (FSE), gradient echo (GRE), 2D and 3D fluid attenuated inversion recovery (FLAIR), 3D susceptibility weighted imaging (SWI). The study was completed with single voxel proton (H) MR spectroscopy (MRS) of a volume of brain white and/or grey matter. MR images were double-checked for structural abnormalities by a child neuroradiologist (RP) and a child neurologist (SF).

### 2.3. Statistical Analysis

Continuous data were expressed as mean ± SD or median ([25–75] percentile) according to variables’ distribution, while categorical data were expressed as frequencies and percentages. The distribution of continuous variables between the two groups (CAS-LI and CAS-LI + CND) was compared using independent Student’s *t*-test or Mann–Whitney nonparametric test, as appropriate. To compare the distribution of categorical data, *χ*^2^ test and Fisher exact test were performed. The pairwise Spearman correlation coefficient was used to describe associations between continuous data.

A cluster analysis was performed to identify possible subgroups within the sample based on the distribution of all the variables considered for the multidimensional assessment of subjects and using an approach specifically dedicated to the analysis of mixed continuous and categorical data. In particular, the adopted method is based on the application of the partitive k-medoid method, which consists of iteratively grouping the most similar units. Given the nature of the variables, the method was applied to the matrix of dissimilarity between the calculated variables using Gower’s distance. The optimal number of clusters was determined based on the silhouette index.

## 3. Results

The sample included 87 boys and 19 girls with CAS (M/F ratio 4.7:1; mean age at the time of assessment: 6.2 years ± 2.2 years). Preperinatal history revealed that none of the children suffered from severe fetal or neonatal complications.

Based on the type of comorbid manifestations, 52 children were assigned to the CAS-LI subgroup and 54 to the CAS-LI + CND subgroup. Table 1 lists their distribution according to the concurrent neurodevelopmental features.

A positive family history for oral/written language disorders was present in 63% of the cases, with a significantly higher percentage of familial cases among children with CAS-LI (74%) as compared to CAS-LI + CND (52%) (*χ*^2^ = 5.434, *p* = 0.020). Only four children had at least one nuclear family member affected by CAS.

Early vocal behavior was reported as abnormal with absent or reduced babbling in 85% of the children and did not differ significantly between the two groups (*χ*^2^ = 2.940, *p* = 0.086). The mean age at first words was 24 ± 11.7 months, without differences between the groups (t = −1.821, *p* = 0.072). Early gross motor development was delayed in 36.5% of the whole sample, with a statistically significant higher frequency in CAS-LI + CND than in CAS-LI (48.1% and 24% respectively; *χ*^2^ = 6529, *p* = 0.011). In particular, the frequency of early gross motor delay was significantly higher in children with DCD (18/22) compared to children without DCD (21/84, *χ*^2^ = 24.202, *p* < 0.001).

### 3.1. Genetic Investigation

CMA analyses identified “causative” CNVs (C-CNVs) in 26 children (24.5%) (see Table 1 and Table 2) with a significantly higher frequency in children with CAS-LI + CND. Most of the C-CNVs were not recurrent, except for 16p11.2 deletion, detected in five cases. Children with C-CNVs showed a significantly lower nonverbal IQ (77.9 ± 22.7 vs. 93.9 ± 20.2, z = −2.629, *p* = 0.009), whereas speech and language scores did not differ significantly between children with C-CNVs and those with N-CNVs and without CNVs (see Supplementary Table S3 for the list of N-CNVs).

### 3.2. Structural Brain MR

Brain MR imaging was normal in 86 children (81.2%). Thirteen cases (12.2%) showed normal variants with no pathogenic significance (i.e., prominent vascular spaces, low position of cerebellar amygdales but no Chiari malformation, small arachnoid cysts, asymmetry of brain ventricles). Seven children (6.6%) showed structural/signal brain abnormalities with uncertain pathogenic significance for CAS in the absence of neurological symptoms or dysarthria (e.g., Chiari 1 malformation, large arachnoid cysts, mega cisterna magna, unilateral focal dysplasia).

Given the lack of evidence in literature of any clear relationship between minor abnormalities and neurodevelopmental disorders, children with normal brain MR and those with minor anomalies were grouped together for statistical analysis.

No statistically significant differences in the distribution of structural/signal brain abnormalities were found either between CAS-LI and CAS-LI + CND (*χ*^2^ = 0.196, *p* = 0.658) (Table 1) or between children with and without C-CNVs (*χ*^2^ = 0.36, *p* = 0.549).

### 3.3. Speech Performances

Overall, speech profiles were characterized by markedly reduced phonetic inventories (mean number of consonants = 11.7 ± 4.9 out of 21), without differences between the two groups (*p* = 0.310). The percentage of inaccurate speech productions in a single-word production test was 69.4% ± 28.5, with 42.4% ± 28.1 of inconsistent errors and no differences between CAS-LI and CAS-LI + CND (*p* = 0.119 and *p* = 0.175 respectively). The mean number of repetitions of the three-syllable nonword sequence/pataka/over 20 s (DDK rate) was 7.8 ± 7.15. It was significantly slower compared to the performance of our reference group of 4.7-year-old TD children and did not differ between CAS-LI and CAS-LI + CND (*p* = 0.295). The mean speech composite severity score was 5.2 ± 1.0, with no difference between the two groups (*p* = 0.169) (see Table 1).

### 3.4. Language Performances

Expressive grammar was impaired in 93% of the children. The comparison between CAS-LI + CND and CAS-LI showed a higher language composite severity score in the former group (*p* = 0.001). In particular, as shown in Table 1, a higher percentage of children with CAS-LI + CND than with CAS-LI had receptive and expressive vocabulary deficits as well as impaired receptive grammar (*p* = 0.024, *p* = 0.011 and *p* < 0.001 respectively), whereas expressive grammar did not differ significantly between the two groups (*p* = 0.713). As expected, children with ID showed a significantly more severe impairment in receptive and expressive vocabulary (*p* < 0.001 and *p* = 0.001 respectively) and in receptive grammar (*p* < 0.001) compared to the children with the other comorbidities (see Table 1).

### 3.5. Correlations between Speech and Language Measures

All speech measures were significantly correlated with each other and with expressive grammar in both groups (See Table 3). In particular, expressive grammar correlated positively with phonetic inventory, DDK rate and intelligibility and negatively with inaccuracy, inconsistency and syllable omissions in both groups. Moreover, in children with CAS-LI a negative correlation emerged between age and the speech composite severity score (r = −0.379), with older children showing higher phonetic inventory (r = 0.462, *p* = 0.001) and DDK rate scores (r = 0.475 *p* < 0.001), together with a lower percentage of inaccurate speech (r = −0.503) and of syllable omissions (r = −0.366). Children with CAS-LI also showed a positive correlation between age and the level of expressive grammar. We did not find statistically significant correlations with age in children with CAS-LI + CND.

### 3.6. Cluster Analysis

**Figure 1:**
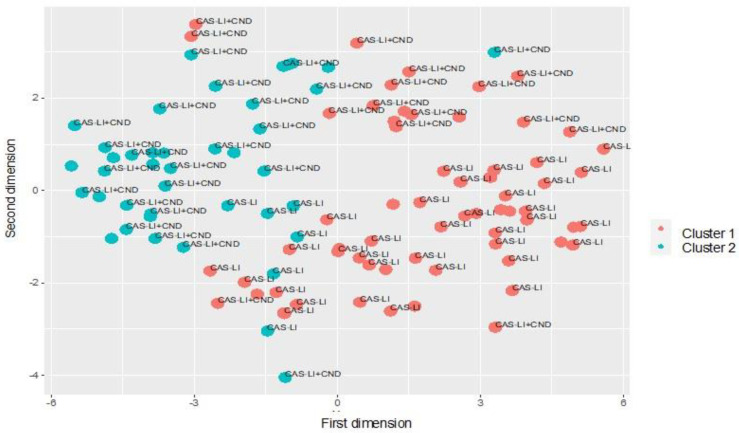
Representation of participants on a bidimensional space according to cluster membership. Each point represents a participant involved in the study. The points are colored according to cluster membership and labelled according to the clinical classification. The distribution of the points suggests that the children belonging to the same cluster are mostly located in the same area. On the other hand, the analysis also reveals a high degree of overlap between the clusters and the clinical classification.

Based on the silhouette values, the solution with two clusters was considered to best fit the data (see Supplementary Figure S1). Cluster analysis of multidimensional variables across the two groups (see Figure 1) showed the presence of a group (Cluster 1, CL1) of 65 children, (46 CAS-LI and 19 CAS-LI + CND) characterized by a less severe speech and language impairment and by more frequent familial antecedents for oral and/or written language disorders. A second cluster (Cluster 2, CL2) included six children with CAS-LI and 35 with CAS-LI + CND, and it was characterized by a more severe speech and language disorder across all the considered measures and by early gross motor delay. Looking at the distribution of the children with CAS-LI and CAS-LI + CND in the two clusters, 88.5% of those with CAS-LI were included in CL1 and 65% of those with CAS-LI + CND in CL2. The distribution of MR findings and genetic variants (CNVs) did not differ significantly between the two clusters (see Table 4).

## 4. Discussion

This paper presents a large-scale clinical investigation of children with comorbid CAS, as well as a contribution to the study of CAS in children acquiring a language other than English.). Our aim was to compare the clinical profiles of children with CAS co-occurring with only language impairment with those who also had other comorbid neurodevelopmental disorders. In the phenotyping of CAS, we attempted to provide a multidimensional description of the disorder across different comorbid conditions by analyzing a set of behavioral and neurobiological features to identify similarities and differences. We a priori separated children with CAS and comorbid complex neurodevelopmental disorders (CAS-LI + CND) from those with CAS associated only to language impairment (CAS-LI). The speech profile did not differ between the two groups, whereas language deficits were more severe and persistent in children with comorbidities. With regard to genetic analyses, about 25% of the participants harbored copy number variations (CNVs) already described in reference to CAS, with a higher frequency in children with complex comorbidities. MR structural/signal alterations were found in a small number of children and were of uncertain pathogenic significance. By grouping the children depending on the severity of the speech and language symptoms, cluster analysis corroborated our choice and offered an alternative point of view to the issue of the clinical heterogeneity of CAS, based on the description of a more functionally oriented profile defined by severity.

### 4.1. CAS Co-Occuring with Complex Neurodevelopmental Disorders

Complex comorbidities occurred in 51% of the children and were mainly represented by ID and DCD, whereas ADHD and ASD, taken together, accounted for 10% of the whole sample. However, only three children with ASD were eligible, being able to complete the full speech and language assessment protocol, so the real relative frequency of CAS in ASD could be effectively higher.

While the association of ADHD with language impairment and reading disorders appears to be well investigated, its co-occurrence with speech sound disorders (SSD) and, particularly with CAS, has been rarely addressed. Studying a large cohort of children with LI and SSD, Lewis et al. [40] found that the severity of language impairment, rather than that of the speech sound disorder, was most predictive of the severity of inattention and hyperactivity/impulsivity. In a more recent study on a small group of children with CAS and comorbid ADHD, LI and a reading disability, Stein et al. [25] reported that ADHD was associated with more severe speech and language profiles. In our study five out of seven children with ADHD clustered together with those with milder speech and language symptoms, suggesting that comorbid ADHD is not necessarily associated with a more severe speech and language disorder.

The high number of children with DCD comorbid to CAS (about 20%) exceeds the current estimates of the prevalence of this disorder in the general pediatric population, most commonly reported to be about 5–6% of the school-aged population [41]. This finding may lend support for the hypothesis of possibly common pathophysiological mechanisms affecting higher order sensory-motor processing [42,43], or to the interpretation of DCD in CAS as the symptom of a widespread multimodal sequencing disorder [44,45,46,47]. Iuzzini-Seigel [48] suggested that the increased risk for motor impairment in children with CAS + LI would stem from “a higher order deficit that mediates cognitive-linguistic and motor abilities”. In our sample we found that DCD frequently co-occurred with early motor delay, while cluster analysis showed no significantly different distribution of DCD in the two identified clusters, suggesting that DCD is not necessarily associated with a more severe speech and language profile.

A diagnosis of ID occurred in about 20% of the participants, and it was mainly of mild degree. As expected, children with ID had a significantly higher language severity score compared to children with other comorbidities due to more severely impaired receptive skills, possibly in relation to lower intellectual abilities. Indeed, at cluster analysis, the majority of the children with ID grouped in the cluster identified by a more severe verbal trait disorder.

### 4.2. Speech and Language Profiles

Descriptive statistics showed that the speech composite severity score and most of the single speech measures did not differ significantly between CAS-LI and CAS-LI + CND, so that the patterns of the speech motor deficits, that represent the core symptoms of CAS, were substantially similar across the comorbidities. Moreover, all speech measures were significantly correlated with each other in both groups. In particular, the percentage of syllable omissions was positively correlated with inaccuracy and inconsistency, and these features were more evident in multisyllabic productions. These findings are in line with the results of some studies on children with CAS, reporting that inconsistency [49] as well as altered movement duration and variability [50] increase in longer syllabic sequences. Moreover, several authors have described how speech and language may interact in complex articulatory contexts or in longer syllable sequences for words and phrases, by taxing the weaker speech motor control of children with CAS and determining breakdowns of various speech parameters [49,50,51].

Also, expressive grammar impairment was present in most of the participants and did not differ between children with CAS-LI and CAS-LI + CND. Moreover, it correlated with the speech measures in both groups, in that more severe motor speech impairment was associated to more limited and altered expressive morpho-syntax.

Expressive vocabulary deficits occurred in children of both groups as well, but with a significantly higher frequency in CAS-LI + CND, despite the similar pattern of speech impairment. Conversely, children with CAS-LI + CND displayed more severe deficits of receptive lexicon and receptive grammar, probably because language comprehension is more vulnerable to deficits across several linguistic and extralinguistic domains.

These results suggest that, as a whole, children with comorbid CAS show a common risk factor for expressive grammar and vocabulary deficits. The interaction between speech motor and language impairment could be more evident in a language such as Italian with longer words and a rich free and bound morphology. However, compared to CAS-LI, in CAS-LI + CND, poorer lexical competence might reflect the involvement of further mechanisms underlying word learning and production such as less background knowledge and poorer cognitive and semantic organization.

Research indicates that in children with CAS lexical acquisition is very slow and atypical starting from the prespeech phases [52,53,54]. Over the course of typical language acquisition, the expansion of expressive lexicon is predicted by the quality and quantity of early vocal and speech behaviors and is paralleled by the progressive improvement of speech motor control [55]. In our sample the signs of an early disruption of vocal and linguistic behaviors were reported by parents of both groups. In particular, babbling was absent or abnormal in 85% of the children, together with delayed vocabulary and grammar milestones that were very common in both CAS-LI and CAS-LI + CND. Absent or sporadic and scarcely variated babbling may reflect disrupted mapping between auditory and motor experiences that is the foundation for speech and language development [56,57]. Therefore, the delay in early vocal and language milestones should be interpreted as the earliest “red flags” for motor speech disorders, indicating difficulties in the construction of articulatory patterns suitable for producing syllables and words [53].

Moreover, cluster analysis showed that the severity of the verbal trait symptoms clustered together with a history of early gross motor delay, particularly in children with CAS-LI + CND, the percentage with delayed early gross motor achievements was significantly higher. The association of late and slow emergence of vocal and verbal behaviors with delayed gross motor milestones should not be underestimated during well-childcare visits. Indeed, it may hint at a globally deviant motor development that could be detected and addressed before the speech and language symptoms of CAS become clinically evident.

Finally, the presence of a significant correlation between age, speech and expressive language performances in CAS-LI, but not in CAS-LI + CND, suggests that speech and language symptoms can persist and are possibly less responsive to treatment in the presence of complex comorbidities. This finding requires further in-depth investigations through longitudinal follow-up studies to see whether, to what extent and in which way comorbid disorders influence the developmental trajectories of the verbal trait symptoms and, therefore, the long-term outcome.

### 4.3. Genetic and Neuroradiological Abnormalities and Their Distribution in CAS-LI and CAS-LI + CND

Demographic data and family history of oral/written language disorders revealed a significantly higher frequency of CAS-LI in males. 

About 24% percent of the children harbored C-CNVs involving genes already described in association with CAS, speech sound disorders or other neurodevelopmental syndromes. The 16p11.2 deletion, the commonest CAS-associated CNV [13,14,15], occurred in five cases. Other rearrangements, detected in single cases, have already been described in CAS [58,59] or in speech and language disorders (7q12 duplication, 15q13 deletion) [60,61]. One child presented a duplication on chromosome 7q35 involving CNTNAP2, a gene candidate for dyslexia, DLD, ASD [10] and CAS [62]. Our data support the known genetic heterogeneity of CAS.

Descriptive statistics showed a significantly higher percentage of C-CNVs in children with CAS-LI + CND and lower nonverbal cognitive skills in the presence of C-CNVs. It is easy to assume that more in-depth genetic studies using WES or WGS will better define different genotype/phenotype associations.

Regarding imaging data, we did not find evidence of the involvement of brain regions typically damaged in adult poststroke apraxia of speech, in agreement with previous literature [21,22]. Unexpectedly, no association emerged between brain abnormalities, which homogeneously occurred in CAS-LI and CAS-LI + CND, and the severity of CAS symptoms. In addition, 13 children presented minor brain anomalies whose prevalence in the general Italian population is currently not exhaustively defined. Brain abnormalities might be the expression of an underlying dysgenesis during brain development yet to be defined and requiring advanced quantitative imaging for further microstructural definition. The intriguing hypothesis of an abnormal organization of the CNS at a microscopic/functional level, suggested in previous studies [63,64,65], deserves further investigation in larger samples, even if we recognize they are hard to perform in clinical settings. Indeed, research in the field of neuroimaging might provide some insights into the pathogenesis of the co-occurring speech and language impairments in CAS. The presence of a partial overlap of anomalies involving motor speech and language areas and circuitries, revealed by morphometric and diffusion MR studies of CAS [21,63,64,65,66], points to possible shared neurofunctional substrates underlying the two disorders.

### 4.4. Limitations and Future Directions

One important limitation was that the number of subjects with CAS associated to ASD and ADHD was too limited to provide a generalizable description of speech motor and language features in these populations. A further limitation was the cross-sectional design of the study that did not allow us to evaluate the developmental trajectories of speech and language in CAS with comorbid neurodevelopmental disorders. Moreover, this paper could not evaluate the impact of comorbid LI in CAS, given that neither children with LI but not CAS, nor with CAS but not LI were included in our experimental sample. Regarding the neurobiological correlates, the type of clinical and instrumental analyses performed in the present study did not allow for an explanation of the neurobiological underpinnings of CAS and its behavioral manifestations. In particular, we could not assess the full genetic landscape in CAS without methodologies of new generation sequencing.

## 5. Conclusions

The results of the present study confirm that CAS is a challenging condition that may be associated with complex neurodevelopmental disorders and, therefore, it needs a multidimensional diagnostic approach and multidisciplinary management. Expressive grammar deficits were a common feature of children in the whole sample and were correlated to substantially similar motor speech core symptoms of CAS across the different neurodevelopmental disorders. One might speculate that motor constraints limiting the construction of speech sequences can have a disruptive effect on expressive grammar, but also that improved language skills can possibly drive better speech sequencing skills. Receptive grammar and lexical deficits were more severe and pervasive in children with complex neurodevelopmental comorbidities, in particular in those with ID.

The presence of concomitant language impairment has important implications for early care and treatment, as the motor speech core symptoms and the language goals should be addressed in an integrated fashion. Moreover, an in-depth characterization of the comorbid neurodevelopmental disorders is of the utmost importance to provide better services and support to the children and their families, and to assist in the quest for a more precise definition of the neurobiological correlates of CAS.

## Figures and Tables

**Table 1:** Comparisons between CAS-LI and CAS-LI + CND.

GENERAL CLINICAL CHARACTERISTICS	CAS-LI (*n*. 52)	CAS-LI + CND (*n*. 54)	
Males:Females ratio	42:10 (4:1)	45:9 (5:1)	*p* = 0.731
Positive Familial History	37/50 * (74%)	28 (52%)	*χ*^2^ = 5.434 *p* = 0.020
Nonverbal IQ	102.10 ± 15.77	79.66 ± 20.97	*p* < 0.001
Motor delay	12 (24%)	26 (48.2%)	*χ*^2^ = 6.529 *p* = 0.011
Abnormal babbling	40 (78.4%)	48 (90.6%)	*χ*^2^ = 2.940 *p* = 0.086
Age at first words	21.6 ± 9.9	25.9 ± 13.12	*t* = −1.821 *p* = 0.072
***COMORBIDITIES*** *(n. of children and percentage)*			
DCD	-	22 (41%)	
ID	-	22 (41%)	
ADHD	-	7 (13%)	
ASD	-	3 (6%)	
***BRAIN MRI***			
Normal or minor anomalies	48 (92%)	51 (94%)	*χ*^2^ = 0.196 *p* = 0.658
Structural abnormalities	4 (8%)	3 (6%)	
***CMA***			
w-CNVs	38 (73%)	27 (50%)	*χ*^2^ = 6.272 *p* = 0.043
N-CNVs	6 (11.5%)	9 (16.7%)	
C-CNVs	8 (15.5 %)	18 (33.35%)	
***SPEECH***			
Phonetic inventory (n. of consonants)	12.2 ± 5.1	11.2 ± 4.6	*t* = 1.021 *p* = 0.310
Inaccuracy (% on productions)	65.02 ± 30.70	73.65 ± 25.69	*t* = −1.577 *p* = 0.119
Inconsistency (% on productions)	38.62 ± 28.91	46.35 ± 27.07	*t* = −1.367 *p* = 0.175
DDK3 (n. of trisyllabic sequences repetitions)	10 [0–14]	9 [0–13]	*Z* = 1.047 *p* = 0.295
Intelligibility score (range 0–5)	2.52 ± 0.96	2.56 ± 0.86	*p* = 0.619
Syllable omissions in words (%)	28.10 ± 33.58 Median 17	45.25 ± 42.11 Median 32	*p* = 0.062
Speech Composite Severity Score (range: 0–6)	5.01 ± 1.14	5.29 ± 0.90	*t* = −1.385 *p* = 0.273
***LANGUAGE***			
Receptive vocabulary (% of subjects with deficient performance)	12/51 (23.5%) **	24 (44.4%)	*χ*^2^ = 5.092 *p* = 0.024
Expressive vocabulary (% of subjects with deficient performance)	24 (46.1%)	38 (70.4%)	*χ*^2^ = 6.399 *p* = 0.011
Receptive grammar (% of subjects with deficient performance)	11 (21.1%)	29/53 (54.7%) **	*p* < 0.001
Complexity of expressive grammar (% of subjects with deficient performance)	48 (92.3%)	51 (94.4%)	*p* = 0.658
Language Composite Severity score (range: 0–4)	2.60 ± 0.93	3.27 ± 0.95	*t* = 3.567 *p* < 0.001

* Data not available (n.a.) for two children; ** Data n.a. for one child; In bold: *p*-value < 0.05; Notes: CAS-LI: childhood apraxia of speech and language impairment; CAS-LI + CND: childhood apraxia of speech, language impairment + complex neurodevelopmental disorders; DCD: developmental coordination disorder; ID: intellectual disability; ADHD: attention deficit hyperactivity disorder; ASD: autism spectrum disorder; CNV: copy number variation; w-CNVs: without CNVs; N-CNVs: noncausative CNVs; C-CNVs: causative CNVs; DDK3: 3 syllable diadochokinetic rate.

**Table 2:** List of causative copy number variants (C-CNVs) detected in our sample.

Patient n.	Chromosome	CMA Findings (hg19)	Size (bp)	Inheritance	Candidate Genes/Loci (Reference)	Disorder
1	11	11p13 deletion	643,641	de novo	CAPRIN1 [36]	CAS-LI
2	4	4q31.1duplication	331,420	maternal	LRBA [36]	CAS-LI + CND (ID)
3	16	16p13.2 duplication	220,893	paternal	ABAT [36]	CAS-LI + CND (ID)
	16	16q23.1 deletion	72,365	maternal	WWOX [36]	
4	8	8p23.1 duplication	366,866	paternal	TNKS [37]	CAS-LI + CND (ID)
5	1	1p34.1 duplication	393,673	paternal	IPP [38]	CAS-LI + CND(ASD)
6	11	11q23.2 duplication	273,131	maternal	NCAM1 [39]	CAS-LI + CND (ADHD)
7	X	Xp11.4 duplication	106,971	maternal	ATP6AP2 [MIM 300423]	CAS-LI + CND (ASD)
8	17	17q12 duplication	1,261,947	paternal	Chromosome 17q12 duplication syndrome [MIM 614526]	CAS-LI
9	15	15q13.2q13.3 deletion	1,496,355	unknown	Chromosome 15q13 deletion syndrome [MIM 612001]	CAS-LI
10	7	7q35 duplication	1350	maternal	CNTNAP2 [36]	CAS-LI + CND (ADHD)
11	16	16p11.2 deletion	524,646	de novo	Chromosome 16p11.2 deletion syndrome [MIM* 611913]	CAS-LI + CND(DCD)
12	16	16p11.2 deletion	446,165	paternal	Chromosome 16p11.2 deletion syndrome [MIM * 611913]	CAS-LI + CND (ADHD)
13	6	6q21 deletion	1,432,328	unknown	6q21 deletion	CAS-LI
14	X	Xq13.3 duplication	18,2919	maternal	ZDHHC15 [MIM * 300577]	CAS-LI + CND (ADHD)
15	16	16p11.2 deletion	545,601	de novo	Chromosome 16p11.2 deletion syndrome [MIM * 611913]	CAS-LI
16	16	16p11.2 deletion	524,999	unknown	Chromosome 16p11.2 deletion syndrome [MIM * 611913]	CAS-LI
17	7	7q11.23 duplication	1,400,000	de novo	Chromosome 7q11 duplication syndrome [MIM * 609757]	CAS-LI
18	4	4q25q26 deletion	5,343,965	de novo	5.3 Mbp deletion	CAS-LI
19	3	3p25.3p26.3 duplication	10,184,886	de novo	10 Mbp duplication, resulting from an unbalanced translocation	CAS-LI + CND(ID)
	21	21q22.3 deletion	3,006,682	de novo	3 Mbp deletion, resulting from an unbalanced translocation	
20	1	1p36 deletion	NA	de novo	Chromosome 1p36 deletion syndrome [MIM * 607872]	CAS-LI + CND(ID)
21	4	4q35.1q35.2 deletion	5,745,530	de novo	5.7 Mbp deletion, resulting from an unbalanced translocation	CAS-LI + CND (ID)
	9	9p24.3p22.1 duplication	18,355	de novo	18 Mbp duplication, resulting from an unbalanced translocation	
22	2	2p16.3 deletion	373,326	de novo	FBXO11 [MIM * 618089]	CAS-LI + CND(ID)
23	22	22q11.21 deletion	1,936,872	unknown	Chromosome 22q11.2 deletion syndrome [MIM * 188400]	CAS-LI + CND(DCD)
24	16	16p13.11 duplication	1144392	unknown	NDE1 [MIM * 614019]	CAS-LI + CND (ID*)*
25	3	3q29 deletion	1,532,486	paternal	Chromosome 3q29 microdeletion syndrome [MIM * 609425]	CAS-LI + CND (DCD)
26	16	16p11.2 deletion	445,805	de novo	Chromosome 16p11.2 deletion syndrome [MIM * 611913]	CAS-LI + CND(DCD)

NA: not available (CMA performed in an external laboratory). Notes: CAS-LI: childhood apraxia of speech and language impairment; CAS-LI + CND: childhood apraxia of speech, language impairment + complex neurodevelopmental disorders; DCD: developmental coordination disorder; ID: intellectual disability; ADHD: attention deficit hyperactivity disorder; ASD: autism spectrum disorder; * MIM: online Mendelian inheritance in man (OMIM) database [35].

**Table 3:** Pairwise Spearman *rho* correlations in CAS-LI and CAS-LI + CND.

CAS-LI	Nonverbal IQ	Age	Age at First Words	Expressive Grammar	Language Composite Severity Score	Phonetic Inventory	Inconsistency	Inaccuracy	Syllable Omissions	DDK3	Intelligibility
Nonverbal IQ	—										
Age	0.157	—									
Age at first words	−0.054	0.073	—								
Expressive Grammar	0.235	0.427 **	−0.223	—							
Language Composite Severity Score	−0.363 *	−0.003	0.124	−0.477 ***	—						
Phonetic Inventory	0.197	0.462 ***	−0.292 *	0.714 ***	−0.301 *	—					
Inconsistency	−0.235	−0.247	0.117	−0.306 *	0.167	−0.418 **	—				
Inaccuracy	−0.384 **	−0.503 ***	0.164	−0.525 ***	0.322 *	−0.618 ***	0.611 ***	—			
Syllable Omissions	−0.297 *	−0.333 *	0.302 *	−0.718 ***	0.459 **	−0.720 ***	0.634 ***	0.675 ***	—		
DDK3	0.360 *	0.475 ***	−0.146	0.510 ***	−0.329 *	0.525 ***	−0.435 **	−0.596 ***	−0.634 ***	—	
Intelligibility	0.201	0.268	−0.015	0.433 **	−0.114	0.461 ***	−0.234	−0.287 *	−0.386 **	0.439 **	—
Speech Composite Severity Score	−0.103	−0.349 *	0.22	−0.619 ***	0.364 **	−0.714 ***	0.431 **	0.591 ***	0.730 ***	−0.634 ***	−0.380 **
Nonverbal IQ	—										
Age	−0.309 *	—									
Age at first words	0.004	0.211	—								
Expressive Grammar	0.317 *	0.19	0.219	—							
Language Composite Severity Score	−0.462 ***	0.024	−0.07	−0.499 ***	—						
Phonetic Inventory	0.237	0.242	0.096	0.536 ***	−0.227	—					
Inconsistency	−0.168	−0.082	0.064	−0.410 **	0.357 *	−0.400 **	—				
Inaccuracy	−0.245	−0.185	−0.012	−0.578 ***	0.409 **	−0.649 ***	0.590 ***	—			
Syllable Omissions	−0.226	−0.135	−0.051	−0.560 ***	0.469 **	−0.472 **	0.720 ***	0.830 ***	—		
DDK3	0.410 **	0.261	0.008	0.630 ***	−0.476 ***	0.468 ***	−0.495 ***	−0.649 ***	−0.764 ***	—	
Intelligibility	−0.027	0.145	−0.029	0.226	0.064	0.436 **	−0.171	−0.361 **	−0.190	0.232	—
Speech Composite Severity Score	−0.106	−0.089	0.236	−0.401 **	0.081	−0.509 ***	0.632 ***	0.653 ***	0.747 ***	−0.498 ***	−0.500 ***

Notes: CAS-LI: childhood apraxia of speech and language impairment; CAS-LI + CND: childhood apraxia of speech, language impairment + complex neurodevelopmental disorders; IQ: intelligence quotient DDK3: 3-syllable diadochokinetic rate. * *p* < 0.05; ** *p* < 0.01; *** *p* < 0.001.

**Table 4:** Cluster analysis: distribution in relation to the clinical subgroups and to the assessed variables.

	Cluster 1 (n = 65)	Cluster 2 (n = 41)	*p*-Value
**CLINICAL SUBGROUPS**			
CAS-LI	46 (88.5%)	6 (11.5%)	**<0.001**
CAS-LI + CND	19 (34.2%)	35 (64.8%)	
**COMORBID DISORDERS** LI-only	46 (70.8%)	6 (14.6%)	**<0.001**
ADHD	5 (7.7%)	2 (4.9%)	0.704
ASD	1 (1.5%)	2 (4.7%)	0.558
ID	2 (3.1%)	20 (48.8%)	**<0.001**
DCD	11 (16.9%)	11 (26.8%)	0.328
**BRAIN MRI**			
Normal	61 (93.8%)	38 (92.7%)	1
Abnormal	4 (6.2%)	3 (7.3%)	
**CMA**			
w-CNVs and N-CNVs	51 (78.5%)	30 (73.2%)	0.687
C-CNVs	14 (21.5%)	11 (26.8%)	
**AGE**	75.9 ± 25.7	81.1±28.6	0.352
**GENDER**			
Male	55 (84.6%)	32 (78%)	0.55
Female	10 (15.4%)	9 (22%)	
**FAMILY HISTORY**			
No	19 (29.7%)	20 (50%)	0.061
Yes	45 (70.3%)	20 (50%)	
**GROSS MOTOR DELAY**			
No	51 (81%)	15 (36.6%)	**<0.001**
Yes	12 (19%)	26 (63.4%)	
**BABBLING**			
Normal	13 (20.7%)	3 (7.5%)	0.159
Atypical	51 (79.7%)	37 (92.5%)	
**FIRST WORDS** **(age in months)**	21.7 ± 10	27.1 ± 13.7	**0.046**
**LANGUAGE**			
***Receptive Vocabulary***			
Normal	56 (87.5%)	13 (31.7%)	**<0.001**
Deficient	8 (12.5%)	28 (68.3%)	
***Expressive Vocabulary***			
Normal	41 (63.1%)	3 (7.3%)	**<0.001**
Deficient	24 (36.9%)	38 (92.7%)	
***Receptive Grammar***			
Normal	56 (86.1%)	9 (22.5%)	**<0.001**
Deficient	9 (13.9%)	31 (77.5%)	
***Expressive grammar***			
Normal	7 (10.8%)	0 (0%)	**0.041**
Deficient	58 (89.2%)	41 (100%)	
**LANGUAGE COMPOSITE SEVERITY SCORE**	2.5 ± 0.9	3.7 ± 0.4	**<0.001**
**SPEECH**			
Phonetic Inventory	12.7 ± 4.9	10±4.5	**0.005**
Inconsistency	32.5 (15–46)	44.5 (34.5–67.5)	**0.008**
Inaccuracy	62.9 ± 29.9	79.8 ± 22.8	**0.001**
Syllable Omissions	13 (0–32)	43 (14.3–97.5)	**<0.001**
DDK3	11.5 (0–15)	0 (0–10.25)	**0.002**
Intelligibility	2.6 ± 0.9	2.5 ± 0.8	0.859
**SPEECH COMPOSITE SEVERITY SCORE**	5.0 ± 1.2	5.4 ± 0.7	**0.015**

Notes: CAS-LI: childhood apraxia of speech and language impairment; CAS-LI + CND: childhood apraxia of speech, language impairment + complex neurodevelopmental disorders; DCD: developmental coordination disorder; ID: intellectual disability; ADHD: attention deficit hyperactivity disorder; ASD: autism spectrum disorder; CNV: copy number variation; DDK3: 3-syllable diadochokinetic rate.

## Data Availability

The data presented in this study are available on request from the corresponding author.

## Supplementary Materials

The following are available online at https://www.mdpi.com/article/10.3390/jpm12020313/s1. Table S1: Speech features of childhood apraxia of speech (CAS) according to the American Speech-Language-Hearing Association (ASHA) consensus and to Strand’s 10-point checklist, Table S2: Speech and language assessment procedures, Table S3: List of noncausative CNVs (N-CNV) in reference to CAS detected in the sample, Figure S1: Silhouette width according to different number of clusters.
