# Supplementary material for: Differences and Commonalities in Children with Childhood Apraxia of Speech and Comorbid Neurodevelopmental Disorders: A Multidimensional Perspective
Source: J Pers Med. 2022 Feb 19;12(2):313. doi: 10.3390/jpm12020313 (PMC8880782; doi:10.3390/jpm12020313)
Supplement: Supplementary file 1 [file jpm-12-00313-s001.zip › Supplementary Table S1.pdf]

Supplementary Table S1: **Speech features of Childhood Apraxia of Speech (CAS) according to the American Speech-Language-Hearing Association (ASHA) consensus and to Strand's 10-point checklist.**

**ASHA: 3 speech features**	1) Inconsistent errors on consonants and vowels in repeated productions of syllables or words.<br>2) Lengthened and disrupted co-articulatory transitions between sounds and syllables.<br>3) Inappropriate prosody, especially in the realization of lexical or phrasal stress.
**Strand's 10-point checklist**	
	1) Vowel or consonant errors including distorted substitutions.<br>2) Intrusive schwa.<br>3) Voicing errors.<br>4) Lexical stress errors or equal stress.<br>5) Syllable segregation.<br>6) Slow rate.<br>7) Difficulty achieving initial articulatory configurations and transitions into vowels.<br>8) Slow DDK rate.<br>9) Groping (articulatory searching prior to phonating).<br>10) Increased difficulty with longer or more phonetically complex words.
