# Supplementary material for: Differences and Commonalities in Children with Childhood Apraxia of Speech and Comorbid Neurodevelopmental Disorders: A Multidimensional Perspective
Source: J Pers Med. 2022 Feb 19;12(2):313. doi: 10.3390/jpm12020313 (PMC8880782; doi:10.3390/jpm12020313)
Supplement: Supplementary file 1 [file jpm-12-00313-s001.zip › Supplementary Table S2.pdf]

Supplementary Table S2. Speech and language assessment procedures.

	Procedure		Reference data	Scoring
	Parental report	Family history, child's pre-, peri- and post-natal clinically significant events, early vocal behavior and language milestones acquisition. Intelligibility of speech to familiar and unfamiliar adults (modified version of the questionnaire reported by Chilosi et al., 2009).		
**Speech Composite Severity Score**	*Phonetic inventory*	Repetition of 21 syllables containing all the Italian consonantal sounds.	40 TD children, mean age=4.7 yrs (SD= 0.47 yrs)	Mean number of phonemes: 19.2 (SD=0.9)
	Word Inaccuracy	46 probe words picture naming test (Chilosi & Podda, in preparation);	40 TD children, mean age=4.7 yrs (SD= 0.47 yrs)	Mean percentage of inaccurate productions: 8.8% (SD=10.7).
	Inconsistent errors on consonants and vowels	Same task as for inaccuracy. Scoring based on the percentage of variable phonetic errors on two repeated productions of the same word.	40 TD children mean age=4.7 (SD=0.47 yrs)	Mean percentage of inconsistent errors: 0.4% (SD= 1.3)
	Syllable omissions	Same task as for inaccuracy. Scoring based on the percentage of omitted syllables in words.	40 TD children mean age=4.7 (SD=0.47 yrs)	Mean percentage of omitted syllables: 0%
	DDK rate (maximum performance task)	Fast repetition of the trisyllabic non-word sequence /pataka/, scored as the number of repeated /pataka/ in 20 sec.	40 TD children (mean age=4.7 yrs (SD=0.9 yrs)	Mean number of repetitions: 23.18 (SD=4.5);
	Intelligibility	Intelligibility in Context Scale (McLeod et al., 2012, Italian version).<br><br>Parental report on the child's intelligibility in different communicative contexts.		Qualitative rating scale ranging from 5 to 1 (5=always, 4= usually, 3= sometimes, 2= rarely, 1= never intelligible)
**Language Composite Severity Score**	*Expressive grammar*	Grid for the Analysis of Spontaneous Speech (GASS) Chilosi et al (2013)	Longitudinal sample: 6 TD children video recorded twice a	12-18 months: Preverbal/Holophrastic level.<br>19–25 months: Presyntactic level, emergence of two- and three-word

			month from 19 to 36 months (Cipriani et al., 1993)<br><br>Cross-sectional sample: 50 t.d. children aged 26-44 mths (Chilosi et al., 2013)	combinations.<br><br>20–26 months: Telegraphic level, emergence of morphosyntactically incomplete subject –verb-object structures<br><br>24–31 months: Grammatical stage 1, full control of morphology in simple sentences<br><br>28–36 months: Grammatical stage 2, production of well-formed both simple and complex sentences
	Receptive grammar	TCGB, Test di Comprensione Grammaticale per Bambini (Grammar comprehension test for children) (Chilosi & Cipriani, 2005)<br><br>TROG-2 Test for Reception of Grammar–Version 2. Dorothy VM Bishop (Italian Version: Suraniti, Ferri & Neri, 2009)	280 ss; age from 3.6 to 8 yrs<br><br>1276 ss (51% F, 49% M); age from 4 to 87 yrs	Standard scores<br><br>Standard scores
	Receptive vocabulary	Test Fonolessicale-TFL (Vicari et al., 2007) and/or<br><br>Peabody Picture Vocabulary Test (PPVT-R - Dunn & Dunn, 1997; Italian version - Stella et al., 2000), depending on the child's age and on the severity of the disorder.	TFL: 240 Italian children from 2.6 to 6 yrs<br><br>PPVT: 2400 Italian children from 3.9 to 11.6 yrs	Percentile scores<br><br>Standard scores
	Expressive vocabulary	Test Fonolessicale-TFL (Vicari et al., 2007) and/or<br><br>One-Word Picture Vocabulary Test (Brizzolara, 1989), depending on the child's age and on the severity of the disorder.	TFL: 268 Italian children from 3 to 6 yrs<br><br>One –Word Picture Vocabulary test: 154 children from 4.6 to 10.8 yrs	Percentile scores<br><br>Separate z-scores for high (52 items) and low (52 items) frequency words.

Abbreviations: TD: Typically Developing; DDK: Diadochokinetic
