# Supplementary material for: Differences and Commonalities in Children with Childhood Apraxia of Speech and Comorbid Neurodevelopmental Disorders: A Multidimensional Perspective
Source: J Pers Med. 2022 Feb 19;12(2):313. doi: 10.3390/jpm12020313 (PMC8880782; doi:10.3390/jpm12020313)
Supplement: Supplementary file 1 [file jpm-12-00313-s001.zip › Supplementary Table S3.pdf]

Supplementary Table S3. List of non causative CNVs (N-CNV) in reference to CAS detected in the sample.

Index case	assembly	band	5'-breakpoint	3'-breakpoint	Lenght (bp)	Dup/Del	Parental origin	Genes in the region*
**Case 29**	hg19	Yp11.2	7241462	8028897	787435	Dup	Father	*PRKY, TTTY12, TTTY16*
**Case 30, 31****	hg19	Xq21.1	77456789	77912844	456055	Dup	Mother	*CYSLTR1, ZCCHC5/RTL3*
**Case 32**	hg19	15q26.1	89349006	89802278	453272	Dup	Father	*ACAN, HAPLN3, MFGE8,* ***ABHD2****, RLBP1,* ***FANCI***
**Case 33**	hg19	5p13.3	33120288	33282513	162225	Del	Adopted	*LINC02160*
	hg19	Yq11.22	24361332	24874501	513169	Del	Adopted	*TTTY5, RBMY2FP, RBMY1F, RBMY1J, PRY, LOC101929148, TTTY6*
**Case 34**	hg19	4q28.1	127722226	127994826	272600	Del	Father	none
**Case 35**	hg19	13q34	114490012	114581677	91665	Del	n.a.	***TMEM255B****, GAS6, GAS6-AS1, GAS6-AS2, LINC00454*
**Case 36**	hg19	9q33.1	118997913	119111534	113621	Dup	Mother	*PAPPA*
**Case 37**	hg19	7q21.3	93355870	93516838	160968	Del	Father	*TFPI2*
	hg19	10q26.2	129846011	129977860	131849	Dup	Mother	***PTPRE****, AS-PTPRE,* ***MKI67***
**Case 38**	hg19	14q31.3	87997862	88365750	367888	Del	Father	*LINC02330*
**Case 39**	hg19	3p21.31	44997133	45313328	316195	Dup	Mother	***ZDHHC3, EXOSC7, CLEC3B****, CDCP1, TMEM158*
**Case 40**	hg19	7q31.1	109574852	109643998	69146	Del	Mother	*EIF3IP1*
**Case 41**	hg19	5p13.3	31484812	31681361	196549	Dup	Mother	*DROSHA, C5ORF22,* ***PDZD2***

Legend: Dup/Del, duplication/deletion; n.a., not available; *, genes expressed in the brain; **, siblings (discordant phenotype)
