# Supplementary figures and images for: Differences and Commonalities in Children with Childhood Apraxia of Speech and Comorbid Neurodevelopmental Disorders: A Multidimensional Perspective
Source: J Pers Med. 2022 Feb 19;12(2):313. doi: 10.3390/jpm12020313 (PMC8880782; doi:10.3390/jpm12020313)

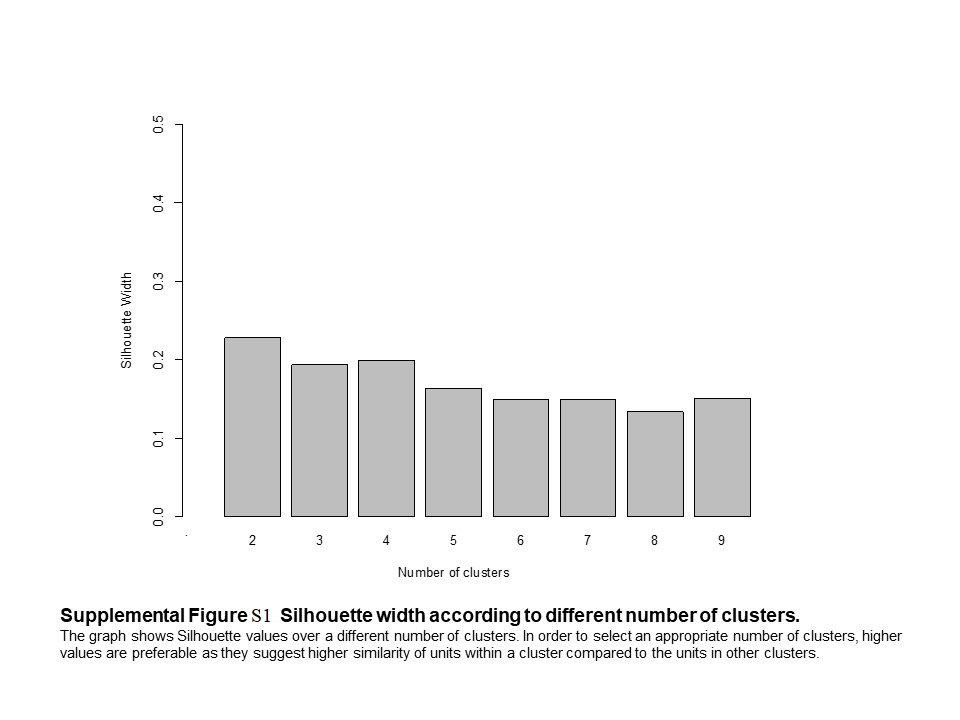

Supplement: Supplementary file 1 [file jpm-12-00313-s001.zip › Supplementary Figure S1.jpg]
